# Sequential Hypertonic-Hypotonic Treatment Enhances Efficacy of Antibiotic against *Acinetobacter baumannii* Biofilm Communities

**DOI:** 10.3390/antibiotics9110832

**Published:** 2020-11-21

**Authors:** Azeza Falghoush, Haluk Beyenal, Douglas R. Call

**Affiliations:** 1 Department of Veterinary Microbiology and Pathology, Washington State University, Pullman, WA 99164-7040, USA; 2 The Gene and Linda Voiland School of Chemical Engineering and Bioengineering, Washington State University, Pullman, WA 99164-7040, USA; beyenal@wsu.edu; 3 Paul G. Allen School for Global Animal Health, Washington State University, Pullman, WA 99164-7090, USA; drcall@wsu.edu

**Keywords:** *Acinetobacter baumannii*, biofilm, osmotic compounds, lipophilicity

## Abstract

Infections with bacterial biofilm communities are highly tolerant of antibiotics. This protection is attributed, in part, to a hydrated extracellular polymeric substance (EPS) that surrounds the bacterial community and that limits antibiotic diffusion. In this study, we evaluated whether it is possible to dehydrate and then re-hydrate a biofilm as a means to increase antibiotic penetration and efficacy. *Acinetobacter baumannii* biofilms (24 h) were exposed to hypertonic concentrations of maltodextrin, sucrose or polyethylene glycol (PEG) as the dehydration step. These biofilms were then washed with deionized water containing 10 times the concentration of antibiotics needed to kill these bacteria in broth culture (50 µg/mL tobramycin, 300 µg/mL chloramphenicol, 20 µg/mL ciprofloxacin or 100 µg/mL erythromycin) as the rehydration step. Biofilms were then harvested, and the number of viable cells was determined. Sequential treatment with PEG and tobramycin reduced cell counts 4 to 7 log (*p* < 0.05) relative to combining PEG and tobramycin in a single treatment, and 3 to 7 log relative to tobramycin treatment alone (*p* < 0.05). Results were variable for other osmotic compounds and antibiotics depending on the concentrations used, likely related to mass and hydrophobicity. Our findings support future clinical evaluation of sequential regimens of hypertonic and hypotonic solutions to enhance antibiotic efficacy against chronic biofilm infections.

## 1. Introduction

Bacterial biofilm is composed of a bacterial community that is embedded in a self-produced matrix that is composed of carbohydrates, proteins and nucleic acids (“extracellular polymeric substance,” or EPS). Once established on biotic and abiotic surfaces, biofilm communities are more resistant to antibiotics compared to planktonic cells. From a clinical perspective *Acinetobacter baumannii* is a biofilm-forming organism that poses serious challenges including with diabetic foot wounds [1,2,3,4,5]. Generally, topical antibiotics are used to treat wound infections, but these treatments suffer from poor antibiotic penetration while potentially adding selective pressure that favors development of antibiotic resistance [6].

The principal mechanism by which the hydrated EPS protects bacteria is via reduced diffusion [7,8], although other mechanisms have also been described [9,10]. These include antibiotic-modifying enzymes, electrostatic interactions, altered microenvironments and slow growth [11,12]. Previous studies suggest that exposing biofilms to hypertonic concentrations of osmotic compounds will significantly change the biofilm structure by reducing biofilm volume and presumable enhancing antibiotic diffusion into biofilm [13] and an atomic force microscopy study of *Acinetobacter baumannii* showed that exposure to a hypertonic solution of maltodextrin collapses both loose and bound EPS [14].

A previous work showed that combining antibiotics with different osmotic compounds helps to kill biofilm communities, but the results were variable depending on the osmotic compound and the mass and lipophilicity of the antibiotics that are used. The authors concluded that combining a low-mass hydrophilic antibiotic with a low-mass osmotic compound was the most effective combination against biofilm of different strains of *A. baumannii* [15].

**Scheme 1:**
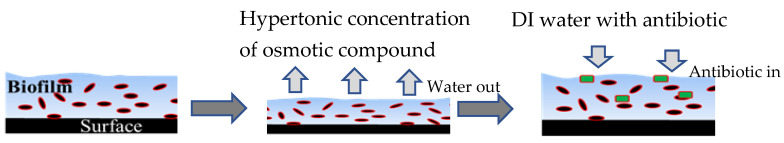
Represents how hypertonic solution of an osmotic compound dehydrates biofilm. De-ionized (DI) water then draws antibiotic into biofilm.

The present study aimed to determine if sequential exposure to hypertonic and hypotonic conditions would increase antibiotic treatment efficiency of *A. baumannii* biofilms. We hypothesized that if a biofilm is first dehydrated by exposure to a hypertonic solution, then rehydration with an aqueous solution containing antibiotic should accelerate penetration and increase the concentration of antibiotic in the biofilm relative to exposure to antibiotic alone. This idea is illustrated in the schematic presentation below (Scheme 1). We also expected that such a process would be enhanced when using antibiotics that are more hydrophilic and comprising smaller molecular mass [15]. To test this hypothesis, *Acinetobacter baumannii* biofilms were dehydrated using hypertonic solutions of maltodextrin, sucrose or polyethylene glycol (PEG). These biofilms were then rehydrated with water containing antibiotics. Biofilms were then harvested, and the efficacy of the treatment was evaluated.

## 2. Results

### 2.1. Sequential Treatment with Hypertonic and Hypotonic Conditions

**Figure 1:**
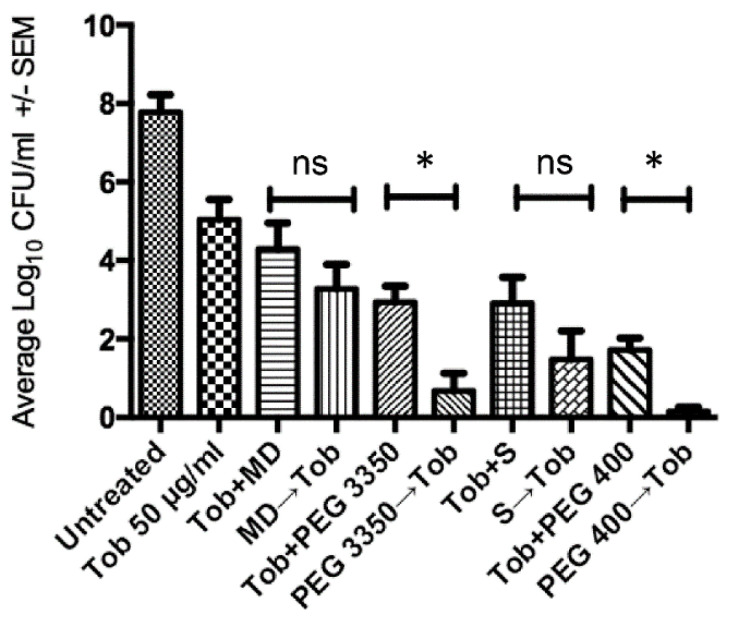
Effects of antibiotic alone, combined (+) or sequential (→) treatment with osmotic compounds maltodextrin (MD), sucrose (S), polyethylene glycol (PEG 3350 or PEG 400) and tobramycin (Tob, 50 µg/mL) against *Acinetobacter baumannii* pre-formed biofilms. Error bars represent the standard error of the means calculated from three biological replicates. Statistical significance was calculated using one-way ANOVA with paired comparisons (Tukey’s test) for individual antibiotics with or without osmotic agent (* *p* < 0.05; ns = not significant).

Tobramycin is a hydrophilic antibiotic (log *p* = −6.2) with a mass of 565 Daltons (g/mol). We first determined if sequential application of hypertonic treatment (maltodextrin, sucrose, PEG) followed by tobramycin in water would enhance the efficacy of the antibiotic against *A. baumannii* biofilm communities compared with antibiotic treatment alone. Treatment of biofilm with tobramycin (10X MIC = 50 µg/mL) inhibited cell recovery by ~3 log (*p* < 0.05) (Figure 1, second column). When simultaneous treatment (osmotic compound plus tobramycin) was compared with sequential treatment, the latter resulted in significantly lower colony forming unite ( CFU) recovery when PEG 3350 or PEG 400 were used (*p* < 0.05), whereas no significant difference was evident for maltodextrin or sucrose (*p* ≥ 0.06). Sequential treatment with PEG 3350 or PEG 400 followed by water with tobramycin reduced recoverable cell counts by approximately 6.8 and 7.9 log CFU/mL, respectively, compared with treatment with tobramycin alone.

### 2.2. Penetration of Biofilms by Antibiotics is Concentration Dependent

**Figure 2:**
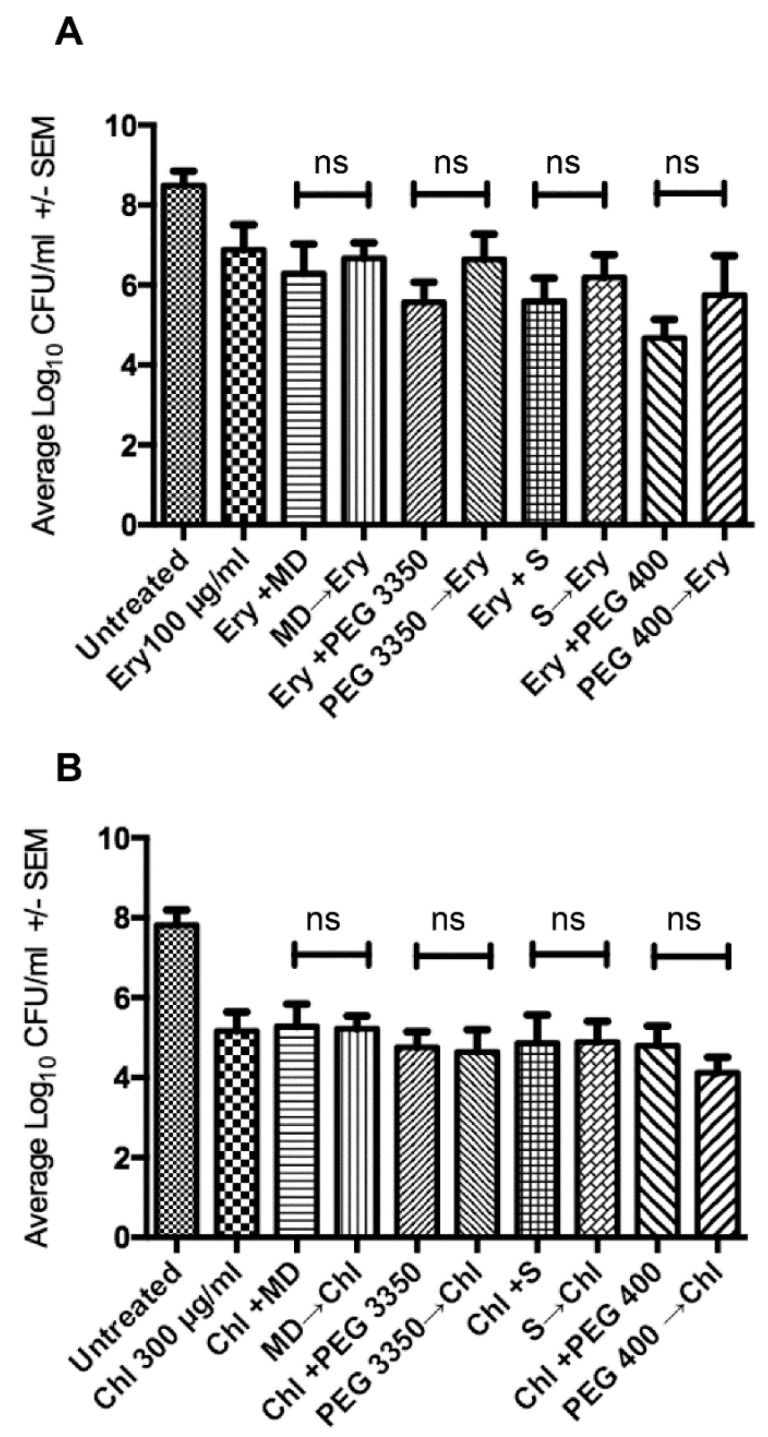
*Acinetobacter baumannii* pre-formed biofilms exposed to antibiotic alone, combined (+) or sequential (→) treatment with osmotic compounds maltodextrin (MD), sucrose (S), polyethylene glycol (PEG 3350 or PEG 400) followed by (**A**) erythromycin (Ery,100 µg/mL) or (**B**) chloramphenicol (Chl 300 µg/mL). Error bars represent the standard error of the means calculated from three biological replicates.

**Figure 3:**
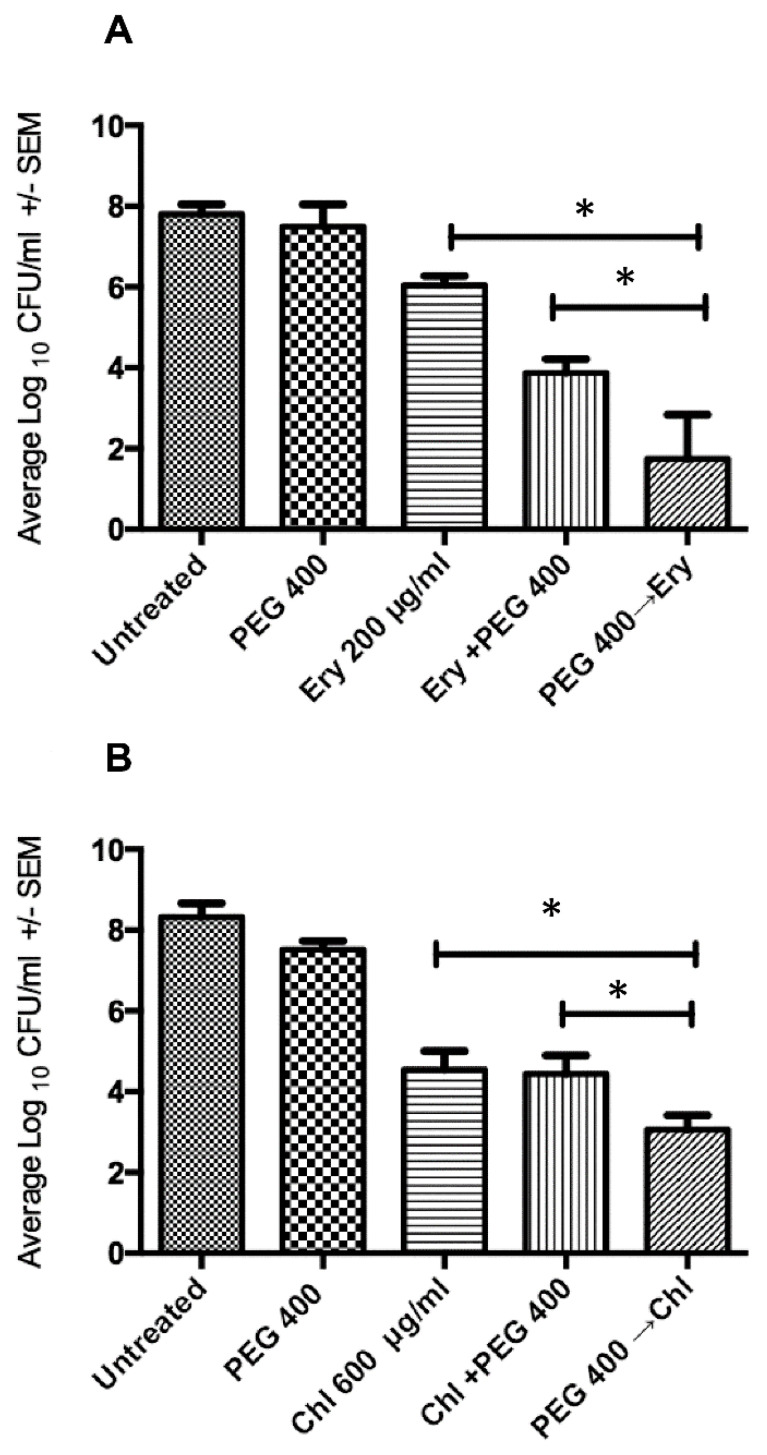
Treatment of *Acinetobacter baumannii* biofilms with individual, combined (+) or sequential (→) treatment of osmotic compound polyethylene glycol (PEG 400) followed by (**A**) erythromycin (Ery, 200 µg/mL) or (**B**) chloramphenicol (Chl 600 µg/mL). Three biological replicates were used to calculate the error bars that represent the standard error of the means (* *p* < 0.05).

Erythromycin (733 Da), chloramphenicol (323 Da), and ciprofloxacin (331 Da) have relatively higher log *p* values (2.7, 1.1, −1.1, respectively) compared to tobramycin and are therefore less hydrophilic. Sequential treatment of osmotic compounds followed by erythromycin (10× MIC = 100 µg/mL) diluted in distilled water did not improve efficacy against biofilm communities relative to erythromycin alone (*p* ≥ 0.4) (Figure 2A). Sequential treatment with chloramphenicol (10× MIC = 300 µg/mL; Figure 2B) or ciprofloxacin (10× MIC = 20 µg/mL; not shown) were also ineffective. Because the mechanism of biofilm penetration is likely to be diffusion and thus concentration dependent, we repeated two of these treatment combinations using 20× MIC concentrations. In both cases, sequential treatment with a hypertonic solution followed by a hypotonic antibiotic solution resulted in a significant reduction in cell recovery (5 to 6 log CFU) compared with antibiotic alone or simultaneous treatment with antibiotic and PEG 400 (*p* < 0.001) (Figure 3A,B).

### 2.3. Sequential Treatment Negates the Limitations of Using Increased Concentration of Osmotic Compounds

**Figure 4:**
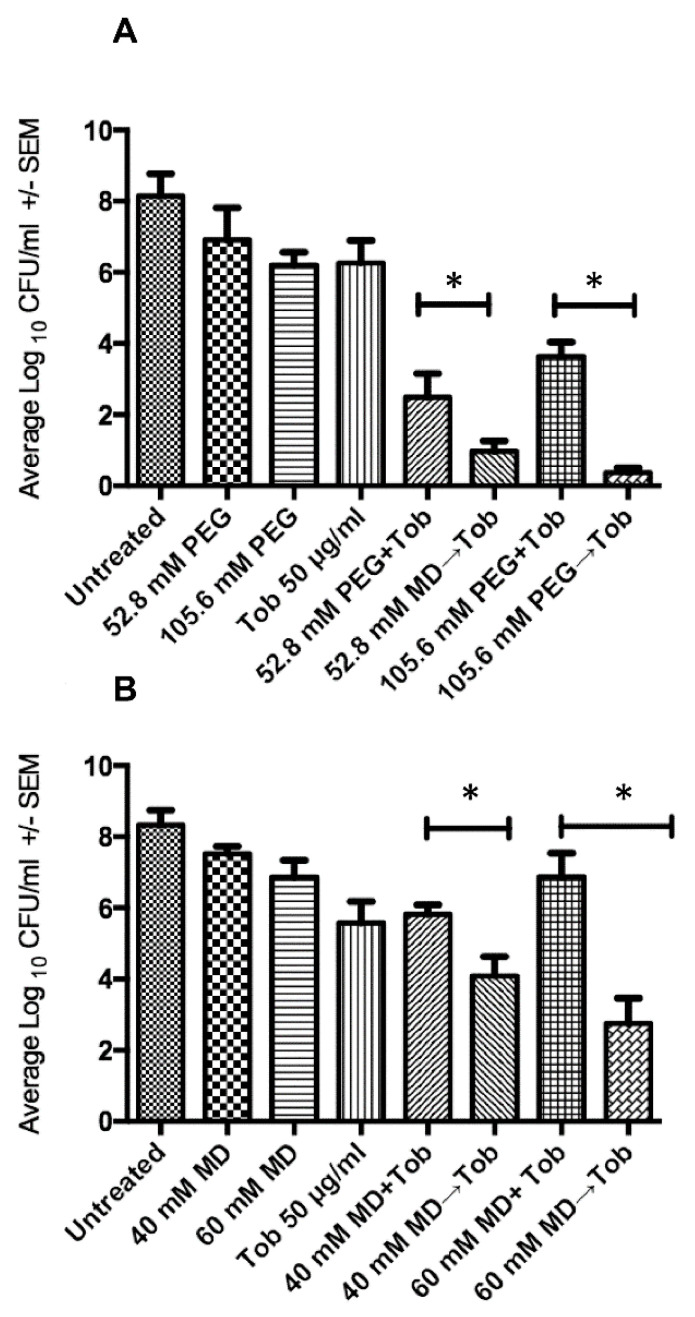
Effects of individual combined (+) or sequential (→) treatment with increased concentrations of osmotic compounds (**A**) maltodextrin (40 or 60 mM) and (**B**) PEG 3350 Da (25.8 or 105.6 mM), followed by tobramycin (Tob, 50 µg/mL). Error bars represent the standard error of the means calculated from three biological replicates. Statistical significance was calculated using a one-way ANOVA and Tukey’s test for tobramycin with or without the osmotic compound (* *p* < 0.05).

Previous work [15] showed that when antibiotics are combined with higher molecular mass osmotic compounds, the solutions can become viscous and this likely reduces antibiotic diffusivity. Herein, we used sequential treatment as a means to circumvent this effect because the viscosity limitations of the hypertonic treatment should have no impact on the penetration of antibiotics during the rehydration step (which relies on an aqueous solution with the antibiotic only). Combined treatment with PEG 3350 and tobramycin produced the expected loss of efficacy between 52.8 mM and 105.6 mM concentrations (Figure 4A), but viscosity limitations that we observed previously in the combinatorial treatment [15] were completely eliminated when using sequential treatment (Figure 4A, last column). Similarly, relative to 40 mM maltodextrin, efficacy of tobramycin declined significantly with 60 mM maltodextrin (*p* < 0.05). This limitation was avoided when the 60 mM maltodextrin was used sequentially with tobramycin (Figure 4B, last column).

## 3. Discussion

Biofilm communities are tolerant of antimicrobials, which is mostly attributed to the hydrated EPS that decreases antibiotic penetration and thus protects biofilm communities [16,17,18]. One strategy to overcome this diffusion barrier is to change the biofilm physical structure by treating the biofilm with hypertonic concentrations of osmotic compounds [13]. These changes in biofilm structure appear to enhance the penetration of antibiotics [19]. Such a treatment will likely work against the bacteria assuming they are normally susceptible to the antibiotic when not protected by a biofilm, and the process works better when the antibiotics have lower mass and are more hydrophilic [15,18,20].

For the current study, we selected 10× of MIC for antibiotic concentration because lower concentrations (3×, 5× and 7×) simply did not show a significant impact against *A*. *baumannii* biofilm communities (data not shown). We did not determine the minimum concentration for antibiotic that could be used when applied sequentially as described in this study. While a 10× MIC concentration may be considered unsafe in many circumstances, our rationale for using a higher concentration is that this will be less of a concern for external wound applications. Further study with these and other antibiotics will be needed to optimize any clinical treatment regimens that are based on sequential treatment of biofilm infections.

For the current study, we examined if this combinatorial treatment could be improved by separating the proposed mechanisms (change in biofilm structure and penetration of antibiotics) into two separate steps. One advantage with doing so is that the process should be less sensitive to the mass and concentration of hypertonic compound. Another advantage is that the sequential treatment may not require a long exposure. Furthermore, previous work showed that only low-molecular mass compounds are effective (<600 Da), probably because high concentrations of high mass osmotic compounds become viscous [15]. Increased viscosity is likely to be counterproductive by reducing the ability of the antibiotic to diffusion efficiently. By separating the dehydration step from the antibiotic application, viscosity is less likely to be an issue making more options available for the hypertonic compounds. 

In this study, combining PEG 3350 or PEG 400 with tobramycin resulted in a 6.8–7.9 log reduction in bacterial counts compared to a 5–6 log reduction when PEG and tobramycin were used simultaneously. PEG is a commonly used polymer in biomedical field, and it is advantageous because of its hydrophilicity, high biocompatibility and decreased interaction with blood components. PEG has demonstrated applications with drug delivery and is generally considered safe for clinical purposes, including for dermatological preparations [21,22,23]. Importantly, while PEG is likely to be an ideal osmotic compound for altering biofilm structure, there is no reason to expect sequential treatment with PEG and an antibiotic to overcome bacteria that are known to be resistant to the antibiotic being used. 

This study highlights the potential of using a sequential hypertonic-hypotonic treatment strategy against wound biofilm infections. Such treatment allows flexibility which hypertonic compound is used, but antibiotics with lower mass and greater hydrophilicity are likely to work better. Work remains to determine how well the proposed strategy will work when used as a clinical treatment, including the duration of application needed to maximize benefits with minimal exposure times. Such a procedure would never be applicable systemically, but our findings suggest that such a strategy should be explored further for treatment of chronic wounds.

## 4. Materials and Methods

### 4.1. Bacterial Strain and Reagents

A clinical isolate of *A. baumannii* (strain ATCC 17978; “Ab17978”; originally isolated from an infant) was used in the present study. The strain was resistant to at least three different categories of antibiotics, including beta lactams. Luria–Bertani (LB, Becton Dickinson and Company, Sparks, MD, USA) agar and broth was used for bacterial culture. Ampicillin, tobramycin, chloramphenicol, erythromycin, ciprofloxacin, maltodextrin, sucrose, 3350 Da polyethylene glycol (PEG), and 400 Da PEG were procured from Sigma-Aldrich (St. Louis, MO, USA). Tobramycin (Tob), chloramphenicol (Chl), erythromycin (Ery) and ciprofloxacin (Cip) were selected for sequential treatment because they represent four different classes of antibiotics with different mass (565, 323, 733, and 331 Da, respectively) and Log P characteristics (−6.2, 1.1, 2.7, and −1.1 units, respectively). Log P is the partial coefficient between *n*-octanol and water, and larger negative values represent more hydrophilic compounds. Log P and mass values used in this study were obtained from PubChem Compound https://www.ncbi.nlm.nih.gov/pccompound/.

### 4.2. Determining Minimum Inhibitory Concentration

The minimum inhibitory concentration (MIC) of *A. baumannii* planktonic culture for each antibiotic was determined based on micro-dilution method as described previously [24]. Briefly, a single colony was inoculated into 3 mL of Mueller-Hinton (MH) media and incubated overnight. Resulting culture was diluted 1:100 in fresh MH media (with or without osmotic compounds) and was added to 96-well plates (100 µL/well). Using multichannel pipette, antibiotic was added to the first set of wells (100 µL/well) and was diluted across wells (1:2). *E. coli* strain K-12 used as negative control. After overnight incubation (37 °C), optical density (600 nm) measurements were collected by using a spectrophotometer. The minimum antibiotic concentration for which optical density measurements were less than two times the standard deviation for the negative control wells was considered the minimum inhibitory concentration. 

### 4.3. Biofilm Preparation

*A. baumannii* biofilms were grown in a test-tube according to previously described methods with minor modifications [25,26]. Briefly, Luria broth (LB) (15 mL) supplemented with 100 µg ampicillin was added to 50-mL conical tubes. Media was inoculated with bacteria from a separate colony of *A. baumannii* that was grown overnight on an LB agar plate. After overnight incubation (37 °C with shaking at 200 rpm), cultures were adjusted to a concentration of ∼1 × 10^8^ colony forming units per ml (CFU/mL) before preparing a 1:100 dilution in fresh LB media. An aliquot (3 mL) of each diluted culture was transferred into 15-mL polystyrene tubes and incubated at 37 °C for 24 h without shaking. This procedure allowed *A. baumannii* to attach to the tube surface and form a biofilm that became visually apparent as a “ring” near the liquid–air interface. The culture supernatant from each tube was then discarded and the attached biofilm was washed three times with sterile distilled water to remove detached cells from the biofilm tube. Biofilms were then considered ready for treatment. 

### 4.4. Biofilm Treatment

Unless indicated otherwise, we treated biofilm communities (in 15 mL tubes as described above) with a concentration of antibiotic that was equivalent to ten-times the MIC needed to kill planktonic cultures of *A. baumannii*. Osmotic compounds were diluted in sterile de-ionized water, including 20-, 40-, or 60-mM maltodextrin, 80-mM sucrose, 6.6-, 52.8-, or 105-mM PEG 3350 (3350 Da), or 26.6-mM of PEG 400 (400 Da). Some of these concentrations were selected based on previous calculations showing that they induce equivalent osmotic pressure [15]. Biofilms were treated independently with an osmotic compound, or an antibiotic, or with a combination of osmotic compound and antibiotic (three independent replicates for each combination). 

For the sequential treatment (dehydration followed by rehydration), the biofilm was first exposed to treatment with an osmotic compound (4 mL) and statically incubated for 1 h at 37 °C. We selected 1 h for convenience, but it is likely that osmosis under these conditions was complete well before this time. The osmotic treatment solution from each tube was then decanted and the biofilm was washed three times with filter-sterilized, deionized water that contained antibiotic. We subsequently added back 4 mL of the antibiotic solution in distilled water and allowed the biofilm to incubate again for 2 h at 37 °C. After treatment, the number of CFU was estimated as follows. Biofilms were first washed with water and harvested by adding LB broth (3 mL) with ~10 sterilized glass beads. Tubes were immediately vortexed for 3 min to dislodge the biofilm and attached bacteria

We found that continuing this procedure longer than 3 min (up to six minutes) did not change the estimated bacterial counts (data not shown) CFU counts of viable cells were then estimated by using serial dilution of the cell suspension (1:10) in LB broth and a 6 × 6 drop-plate protocol [27]. The same protocol was used for controls except no antibiotics or osmotic agent was added.

### 4.5. Statistical Analysis

The bacterial counts were log-transformed prior to analysis. The effect of different treatments on CFU counts was evaluated by using ANOVA and a Tukey multiple-pairwise comparisons test (Sigma Plot, ver.11.0). 

## References

[1] Lee K., Yong D., Jeong S.H., Chong Y. (2011). Multidrug-resistant *Acinetobacter Spp.*: Increasingly problematic nosocomial pathogens. Yonsei Med. J..

[2] Howard A., O’Donoghue M., Feeney A., Sleator R.D. (2012). *Acinetobacter Baumannii*: An emerging opportunistic pathogen. Virulence.

[3] Dijkshoorn L., Nemec A., Seifert H. (2007). An increasing threat in hospitals: Multidrug-resistant *Acinetobacter Baumannii*. Nat. Rev. Microbiol..

[4] Longo F., Vuotto C., Donelli G. (2014). Biofilm Formation in *Acinetobacter Baumannii*. New Microbiol..

[5] Peleg A.Y., Seifert H., Paterson D.L. (2008). *Acinetobacter Baumannii*: Emergence of a successful pathogen. Clin. Microbiol. Rev..

[6] Ciofu O., Rojo-Molinero E., Macià M.D., Oliver A. (2017). Antibiotic treatment of biofilm infections. APMIS.

[7] Gordon C.A., Hodges N.A., Marriott C. (1988). Antibiotic interaction and diffusion through alginate and exopolysaccharide of cystic fibrosis-derived *Pseudomonas Aeruginosa*. J. Antimicrob. Chemother..

[8] Stewart P.S. (1996). Theoretical aspects of antibiotic diffusion into microbial biofilms. Antimicrob. Agents Chemother..

[9] Van Acker H., Van Dijck P., Coenye T. (2014). Molecular mechanisms of antimicrobial tolerance and resistance in bacterial and fungal biofilms. Trends Microbiol..

[10] Taff H.T., Mitchell K.F., Edward J.A., Andes D.R. (2013). Mechanisms of candida biofilm drug resistance. Future Microbiol..

[11] Hall C.W., Mah T.F. (2017). Molecular mechanisms of biofilm-based antibiotic resistance and tolerance in pathogenic bacteria. FEMS Microbiol. Rev..

[12] Davenport E.K., Call D.R., Beyenal H. (2014). Differential protection from tobramycin by extracellular polymeric substances from *Acinetobacter Baumannii* and *Staphylococcus Aureus* biofilms. Antimicrob. Agents Chemother..

[13] Kiamco M.M., Atci E., Khan Q.F., Mohamed A., Renslow R.S., Abu-Lail N., Fransson B.A., Call D.R., Beyenal H. (2015). Vancomycin and maltodextrin affect structure and activity of *Staphylococcus Aureus* biofilms. Biotechnol. Bioeng..

[14] Deliorman M., Gordesli Duatepe F.P., Davenport E.K., Fransson B.A., Call D.R., Beyenal H., Abu-Lail N.I. (2019). Responses of *Acinetobacter Baumannii* bound and loose extracellular polymeric substances to hyperosmotic agents combined with or without tobramycin: An atomic force microscopy study. Langmuir.

[15] Falghoush A., Beyenal H., Besser T.E., Omsland A., Call D.R. (2017). Osmotic compounds enhance antibiotic efficacy against *Acinetobacter Baumannii* biofilm communities. Appl. Environ. Microbiol..

[16] Singh R., Sahore S., Kaur P., Rani A., Ray P. (2016). Penetration barrier contributes to bacterial biofilm-associated resistance against only select antibiotics, and exhibits genus-, strain- and antibiotic-specific differences. Pathog. Dis..

[17] Flemming H., Wingender J. (2010). The biofilm matrix. Nat. Rev. Microbiol..

[18] Olsen I. (2015). Biofilm-specific antibiotic tolerance and resistance. Eur. J. Clin. Microbiol. Infect. Dis..

[19] Kiamco M.M., Mohamed A., Reardon P.N., Marean-Reardon C.L., Aframehr W.M., Call D.R., Beyenal H., Renslow R.S. (2018). Structural and metabolic responses of *Staphylococcus Aureus* biofilms to hyperosmotic and antibiotic stress. Biotechnol. Bioeng..

[20] Stewart P.S. (2002). Mechanisms of antibiotic resistance in bacterial biofilms. Int. J. Med. Microbiol..

[21] Jang H.J., Shin C.Y., Kim K.B. (2015). Safety Evaluation of Polyethylene Glycol (PEG) compounds for cosmetic use. Toxicol. Res..

[22] Knop K., Hoogenboom R., Fischer D., Schubert U.S. (2010). Poly(Ethylene Glycol) in drug delivery: Pros and cons as well as potential alternatives. Angew. Chem. Int. Ed..

[23] Fruijtier-Pölloth C. (2005). Safety assessment on Polyethylene Glycols (PEGs) and their derivatives as used in cosmetic products. Toxicology..

[24] Andrews J.M., Andrews J.M. (2001). Determination of Minimum Inhibitory Concentrations. J. Antimicrob. Chemother..

[25] Abdi-Ali A., Hendiani S., Mohammadi P., Gharavi S. (2014). Assessment of biofilm formation and resistance to imipenem and ciprofloxacin among clinical isolates of *Acinetobacter Baumannii* in tehran. Jundishapur J. Microbiol..

[26] Pour N.K., Dusane D.H., Dhakephalkar P.K., Zamin F.R., Zinjarde S.S., Chopade B.A. (2011). Biofilm formation by *Acinetobacter Baumannii* strains isolated from urinary tract infection and urinary catheters. FEMS Immunol. Med. Microbiol..

[27] Chen C.Y., Nace G.W., Irwin P.L. (2003). A 6x6 drop plate method for simultaneous colony counting and MPN enumeration of campylobacter jejuni, listeria monocytogenes, and escherichia coli. J. Microbiol. Methods.

